# Digital motor markers for early autism detection: promise, pitfalls, and a path to clinics

**DOI:** 10.3389/fpsyt.2025.1720138

**Published:** 2025-12-01

**Authors:** Ileana Scarcella, Simona Campisi, Grazia Serena Previti, Roberta Bruschetta, Assunta La Corte, Gennaro Tartarisco, Giovanni Pioggia, Flavia Marino

**Affiliations:** 1 Institute for Biomedical Research and Innovation (IRIB), National Research Council of Italy (CNR), Messina, Italy; 2 Faculty of Psychology, International Telematic University Uninettuno, Roma, Italy

**Keywords:** autism, motor biomarkers, kinematic analysis, digital phenotyping, early detection

## Introduction

Autism spectrum disorder (ASD) is a complex neurodevelopmental condition that is commonly characterized by repetitive behaviors, limited interests, and difficulties in communication and social interaction (1). Motor system abnormalities, which are frequently overlooked or underestimated, are becoming increasingly recognized as potential biomarkers, even though basic socio-communicative features remain the standard diagnostic criteria (2). Among the numerous motor challenges that are commonly observed and have a cascading impact on cognitive and socio-emotional development are postural control, bilateral coordination, fine motor skills, and motor imitation (3). Recent evidence indicates that infants later diagnosed with ASD exhibit distinct early motor trajectories when compared to typically developing infants (4). Relevant differences in movement activity and altered motor profiles can be detected between 6 and 12 months of age (5–7), before the onset of clear socio-communicative symptoms, making them powerful, non-verbal indicators of atypical neurodevelopmental trajectories.

Recent advances in motion-capture, wearable sensors, and computer-vision (CV) systems allow researchers to utilize kinematic motion analysis and digital phenotyping to derive digital motor markers (DMMs). A recent systematic review by Simeoli et al (10). synthesized studies applying machine learning to motion‐analysis data for early detection of ASD, demonstrating that motion patterns could classify autism with accuracies comparable to traditional tools, while also highlighting important methodological challenges (8). These markers include specific kinematic features, such as trajectory linearity, speed, acceleration and direction changes, as recorded during children’s simple motor tasks (9, 10). Although DMMs have a lot of promise for objective, early ASD detection, this paper argues that before they can be applied in clinical settings, major challenges with standardization, interpretability, and validation need to be addressed. We propose a way forward for the clinical translation of movement data into usable indicators for timely recognition and personalized early intervention.

While many studies have interpreted early motor differences in autism as a developmental delay, a growing body of evidence suggests that they may instead reflect atypical maturation of sensorimotor circuits. This distinction is crucial, as it reframes motor atypicalities not as transient deficits but as enduring traits that shape neurodevelopment across the lifespan. This opinion paper adopts this latter perspective, arguing that motor markers should be conceptualized as lifespan-relevant indicators of neurobiological divergence rather than early, transient anomalies.

## Neurobiological and clinical significance of motor atypicalities

According to the literature, significant motor anomalies may be present in between 50% and 88% of autistic children, suggesting that these dysfunctions may not merely be secondary correlates. A growing body of research suggests that motor features, which emerge early in development and are understudied components of the neurobiological profile of autism, may be foundational for some autistic phenotypes (3). According to a range of neurobiological theories (11) early developmental dysfunctions of the brainstem and other subcortical structures involved in sensorimotor regulation are the cause of motor abnormalities in autism. Through compensatory cortical mechanisms, these impairments interfere with primary sensorimotor regulation, which in turn affects the development of socio-communicative skills. Emerging evidence from neuroimaging studies, brainstem pathology, animal models, and longitudinal research suggests that motor difficulties are not mere comorbidities but rather represent an early and central feature of autistic neurodivergence (12–14).

Clinically, these variations in motor function could be parallel indicators or possible precursors of abnormal neural development. The sensorimotor basis that regulates higher-order social and cognitive functions may be disrupted by impairments in basic motor abilities (15). The development of complex social and communicative skills may be affected indirectly by autistic children’s subtle delays in imitation, gesture production, and object manipulation, for example, which may restrict opportunities for social engagement and joint attention (16). Thus, the incorporation of kinematic analysis into early developmental evaluations provides a unique opportunity to find risk indicators that are both objectively quantifiable and based in neurobiology, potentially changing the paradigm for autism early detection.

Importantly, atypical kinematic signatures are not confined to infancy or childhood. Recent lifespan studies have demonstrated that distinctive patterns of motor planning and execution persist into adolescence and adulthood, supporting the view that these features reflect stable neurodevelopmental traits rather than simple developmental delay (17–19). Such evidence underscores the need to examine DMMs as longitudinal markers of atypical sensorimotor maturation.

## The promise: digital motor markers for high-resolution phenotyping

Beyond the subjective limitations of conventional behavioral assessments or parent-report questionnaires, kinematic motion analysis is a very promising and objective technology for the early detection of ASD. Using high-resolution estimation of movement variables like position, velocity, and acceleration, this method reveals micro-level deviations that are frequently disregarded in aggregated clinical scores (20, 21).

Researchers can record movement trajectories during both structured and naturalistic tasks with millisecond precision by using technologies like wearable sensors, computer vision technologies, and 3D motion capture systems (22, 23). Even during naturalistic interactions or imitation tasks, these techniques allow for the real-time collection of highly detailed kinematic data, which enables the detection of even the smallest motor atypicalities and accurate descriptions of intra- and inter-individual variability (24).

Within this framework digital motor markers (DMMs) are defined as objective kinematic characteristics that represent particular aspects of motor coordination, control, and planning. Recent work, including that by Torres (25) and Simeoli et al. (10), has grounded the interpretation of these markers within the predictive coding framework, highlighting how atypical motor variability may reflect alterations in sensorimotor integration. This conceptual framing provides a systematic link between individual DMMs and underlying neurobiological mechanisms, emphasizing that motor atypicalities in ASD can be understood as predictive coding disruptions rather than simple delays.

The following are typical examples: dyadic visuomotor synchrony in parent-child play (evaluating temporal coordination in social interaction); movement smoothness or jerk (capturing motor control efficiency); trajectory curvature or path length ratio in goal-directed pointing (indicating online correction demands); tapping-task variability (reflecting rhythmic stability); and postural sway measures during quiet stance (probing balance-control mechanisms) (25, 26).

To aid researcher in navigating the diversity of digital motor markers, **Table 1** summarizes key DMMs along with their associated evidence and relevant developmental stages.

**Table 1:** Representative Digital Motor Markers (DMMs) and related evidence.

Digital motor marker (DMM)	Description kinematic feature	Motor domain assessed	Typical age range studied	Evidence
Movement smoothness (jerk, spectral arc length)	Quantifies fluency and coordination of motion through velocity or acceleration profiles	Motor control efficiency Online correction ability	Infancy Childhood	Anzulewicz et al., 2016 (9)
Trajectory curvature Path length ratio	Measures deviation of limb trajectory from an optimal straight path	Motor planning Predictive coding Visuomotor control	Infancy Early childhood	Torres, 2013 (25); Bäckström et al., 2021 (41)
Temporal coupling Synchrony in dyadic interaction	Degree of temporal alignment between child and partner movements	Social motor coordination Imitation	Toddlerhood Middle childhood	Zampella et al., 2020 (26); Fulceri et al., 2018 (16)
Movement variability (intra-individual coefficient of variation)	Quantifies stability and reproducibility of repeated actions	Motor consistency Adaptive control	Childhood Adolescence	Anzulewicz et al., 2016 (9); Cook et al., 2013 (43)
Postural sway (center-of-pressure displacement)	Reflects micro-adjustments required to maintain balance	Postural control Proprioception	Childhood Adulthood	Fournier et al., 2010 (34); Kaur et al., 2018 (3)
Dragging-task kinematics (velocity, acceleration, trajectory features)	Quantifies fine motor control via kinematic features from a dragging task	Fine motor control Visuomotor coordination Sensorimotor integration	Childhood	Simeoli et al., 2021 (10)
Eye-movement metrics (fixation stability, saccadic latency)	Captures gaze control and visuomotor coupling	Visual attention Oculomotor control	Infancy Adulthood	Avni et al., 2021 (31); Ziv et al., 2024 (32)

The diagnostic potential of these digital features has already been shown in some studies. In the first and second years of life, Bhat et al. (21) found systematic delays in different kinds of behaviors (such as reaching, clapping, pointing, etc.), while Anzulewicz and colleagues (9) used tablet-based motion data to differentiate children with ASD from typically developing peers with over 90% accuracy. In the same direction, Zampella et al. (26) discovered that during imitation tasks, children with autism exhibit variations in intrapersonal and interpersonal coordination (around 82% accuracy). These results contribute to the idea that motor profiles could be helpful indicators of atypical neurodevelopment. Inexpensive inertial sensors and computer vision/pose-estimation techniques, when applied to standard video from tablets or smartphones, provide non-invasive methods suitable for children, making them scalable and viable for community-based screening and optimizing ecological validity (27, 28).

Kinematic data’s potential for automated screening and customized evaluation is further increased by its integration with machine learning (ML) and artificial intelligence (AI)-driven analytics (10). Research employing computer-vision tools such as OpenPose or MediaPipe has demonstrated that automated pose estimation can consistently extract kinematic features from regular video recordings, with promising accuracy levels that differentiate ASD from typical development based on motor patterns (29, 30).

Beyond hand and body movements, eye-movement dynamics represent another promising but underexplored source of motor information. Studies have revealed altered oculomotor variability and gaze randomness in autistic individuals during visual exploration tasks, suggesting that eye-movement parameters could serve as complementary DMMs that capture visuomotor coordination and attentional control (31, 32).

Together, these results emphasize the potential of DMMs for high-resolution digital phenotyping, comparable to the way digital tools have transformed diagnostics in domains such as Parkinson’s disease (33). This technology bridges the gap between neurobiology, behavior, and clinical practice by converting subtle motor atypicalities into reliable, quantifiable DMMs.

## The pitfalls: challenges to standardization and clinical validation

Even though DMMs show promise in diagnosing autism, several significant challenges need to be resolved before they can be successfully employed in clinical settings.

First of all, because the autism spectrum is inherently heterogeneous and encompasses a broad range of traits, comorbidities, and developmental trajectories, the high variability of motor patterns within the spectrum restricts interpretation (34). Coordination and smoothness are examples of motor characteristics that can vary between people and even within the same person depending on the context. Furthermore, because of the rapid changing of these patterns across developmental stages, analysis requires standardization specific to age, sex, and experience. For instance, a DMM sensitive to a specific reach-to-grasp anomaly at 12 months may be irrelevant at 3 years. This requires DMMs to be constantly calibrated across the lifespan, which demands longitudinal data that are currently scarce.

Context dependence of motor behavior is a second major concern, emphasizing that environmental factors, including task design, emotional states, and physical and social contexts, affect kinematic parameters (35). Moreover, ecological validity warrants closer attention. Most studies rely on structured laboratory tasks, which may not fully capture the richness and variability of spontaneous movements in naturalistic settings. Incorporating tasks that reflect everyday motor behaviors can improve the translational relevance of DMMs and ensure that findings generalize beyond controlled experimental conditions.

Third, there are still significant technical obstacles linked to data quality and standardization. Protocol and data collection inconsistencies arise from the current use of many different and costly technologies, such as wearable sensors, computer vision pipelines, and 3D motion capture. This discrepancy restricts the creation of large and representative datasets required for efficient AI model training by preventing comparison of datasets across various studies (28). Because of this, a lot of research uses small, non-representative sample sizes, which increases the risk of overfitting and reproducibility problems in larger, more diverse populations.

A critical limitation for clinical translation is the current absence of large normative datasets covering different ages and sexes. Without age- and sex-specific benchmarks, it remains difficult to determine whether deviations in DMMs represent genuine atypicality or normative developmental variability. Establishing cross-sectional and longitudinal normative reference databases should therefore be a top research priority before clinical implementation.

When using video and sensor-based recordings in home or educational settings, digital phenotyping brings new ethical and clinical challenges, particularly with regard to data privacy and security. Establishing transparent informed consent frameworks that respect the autonomy of children and their families is critical (36).

Additionally, when trained on data restricted to particular populations (e.g., white males, high-resourced clinics), AI models in healthcare face the risk of algorithmic bias and overdiagnosis, which could misclassify children from diverse backgrounds and worsen already-existing disparities (37).

Lastly, the interpretability of DMMs is critical to their clinical efficacy; without transparent and explainable models, clinicians may find it difficult to extract useful information. For these models to be dependable instruments that complement, rather than replace, professional judgment in autism diagnosis, rigor must be ensured.

## Discussion

The real challenge today lies in translating the potential of emerging technologies into accessible, reliable, and clinically relevant tools that can be stably integrated into early assessment and translational research practice. Only through this transition can the richness of motor data be fully leveraged, transforming it into effective indicators for the timely recognition of autism and the design of personalized interventions to support child development.

Despite growing scientific interest in motor dysfunctions in ASD, current diagnostic and clinical frameworks remain heavily focused on socio-communicative domains, often neglecting the crucial role of motor systems in neurodevelopment. Although motor difficulties are not included in the official diagnostic criteria, they represent a significant and potentially predictive component of the autistic phenotype (6). However, motor impairments are not universal across the spectrum. Subgroups of autistic individuals display relatively preserved motor coordination, suggesting the existence of multiple motor phenotypes or endophenotypes within ASD (38). Recognizing this heterogeneity is key to refining both the theoretical understanding and clinical use of DMMs. In particular, detailed analysis of fine arm and hand movements remains underexplored; most studies are limited to coarse measures such as general motor delays (3, 39, 40) or simple temporal differences (34), thereby missing valuable information on the quality and complexity of motor behavior. However, kinematic analysis has repeatedly demonstrated that children with ASD display unique patterns in their movement planning, execution, and modulation, suggesting early disruptions in predictive coding and sensorimotor integration (24, 25, 41–43).

According to these results, DMMs - quantitative features derived from wearable sensors or computer-vision systems – have a lot of potential as objective, high-resolution, and non-invasive biomarkers that can enhance and complement current behavioral assessments. However, future research must prioritize standardizing acquisition protocols (task design, sensor placement, sampling frequency, and duration) and ensuring cross-site reproducibility through harmonized datasets and open methodological reporting in order to achieve real-world clinical impact. To ensure the generalizability, robustness, and equity of the derived digital motor markers, extensive, multisite validation studies with age- and sex-stratified analyses are necessary.

Furthermore, to guarantee that models exhibit genuine clinical utility rather than just algorithmic performance, validation must go beyond basic accuracy and include metrics like Area Under the Curve (AUC), Positive Predictive Value (PPV), Negative Predictive Value (NPV), and calibration. DMM-based outputs must be clinically interpretable in order to be successfully incorporated into primary care, and researchers must also create safe, ethical guidelines for data governance and privacy. In order to ensure accessibility outside of specialized research centers, DMM-based workflows must also be affordable, practical, and scalable in community and primary-care settings.

To advance toward clinical translation, future work should follow a structured roadmap encompassing: (i)The establishment of normative lifespan datasets; (ii)Methodological standardization across laboratories; (iii)Validation of interpretability and fairness in AI-driven models; (iv) Integration of DMM-based tools into accessible, ethically governed clinical workflows.

Such coordinated efforts will allow DMMs to evolve from promising research metrics into clinically actionable biomarkers.

Importantly, this work goes beyond descriptive characterization of motor differences by proposing a conceptual framework that situates DMMs as lifespan-relevant markers of neurobiological divergence. By linking fine-grained kinematic profiles to predictive coding and sensorimotor integration, we provide a structured perspective that can guide both research and clinical translation.

Finally, recognizing the diagnostic and theoretical value of motor signals represents one of the most promising frontiers in autism research. In addition to improving early diagnosis, combining technological innovation and clinical expertise allows us to better understand the neurobiological mechanisms underlying autism and create individualized, developmentally appropriate interventions. Such integration could contribute to developing more effective care pathways, ultimately enhancing the quality of life for individuals on the spectrum and their families.
